# Extracorporeal Cardiopulmonary Resuscitation for 78‐min Cardiac Arrest due to Suspected Cerebral Venous Sinus Thrombosis

**DOI:** 10.1155/crcc/7873285

**Published:** 2025-12-12

**Authors:** Dan Wu, Haidong Qin

**Affiliations:** ^1^ Department of Emergency Medicine, Nanjing First Hospital, Nanjing Medical University, Nanjing, Jiangsu, China, njmu.edu.cn

**Keywords:** bilateral decompressive craniectomy, cardiac arrest, cerebral venous sinus thrombosis, extracorporeal cardiopulmonary resuscitation (ECPR)

## Abstract

A 37‐year‐old man had a cardiac arrest. His colleagues started CPR and called an ambulance to our emergency department. The ECMO team assessed him and initiated ECPR. Coronary, left ventricular, and pulmonary angiography showed no abnormalities. The patient had pupil dilation, prompting a bilateral decompressive craniectomy under heparin‐free ECMO support. Postoperatively, pupils remained dilated until cerebral angiography revealed venous sinus thrombosis, prompting anticoagulation therapy, which led to pupil constriction and partial neurological recovery. The patient was weaned off ECMO and was transferred to a rehabilitation hospital. Successful resuscitation after prolonged cardiac arrest using ECPR, bilateral decompressive craniectomy, and CVST treatment is rare.

## 1. Introduction

The global annual incidence rate of cardiac arrest (CA) is estimated to range from 35 to 55 cases per 100,000 individuals, resulting in over 5 million deaths each year. Notably, sudden cardiac death accounts for more than 50% of all cardiovascular disease‐related deaths and approximately 20% of total global mortality [1]. Globally, the average survival rate for out‐of‐hospital CA remains below 10%, whereas in China, this figure is as low as 1.2%. In contrast, under professional emergency care settings, the survival rate can increase to as high as 80% [2]. Cardiopulmonary resuscitation (CPR) is the most effective intervention for patients with CA; however, its overall success rate remains as low as 9.9% [3]. Over the past decade, the integration of extracorporeal membrane oxygenation (ECMO) with CPR, known as extracorporeal cardiopulmonary resuscitation (ECPR), has substantially increased the survival rates of CA patients and improved their neurological outcomes [4, 5]. The causes of CA are multifactorial and heterogeneous. The application of ECPR not only provides a critical time window for addressing the underlying cause but also facilitates the recovery of neurological function by creating optimal conditions. Here, we present a case of a patient who experienced prolonged CA for 78 min, underwent ECPR, achieved partial neurological recovery, and was subsequently discharged from the hospital.

## 2. Case Presentation

**Figure 2:**
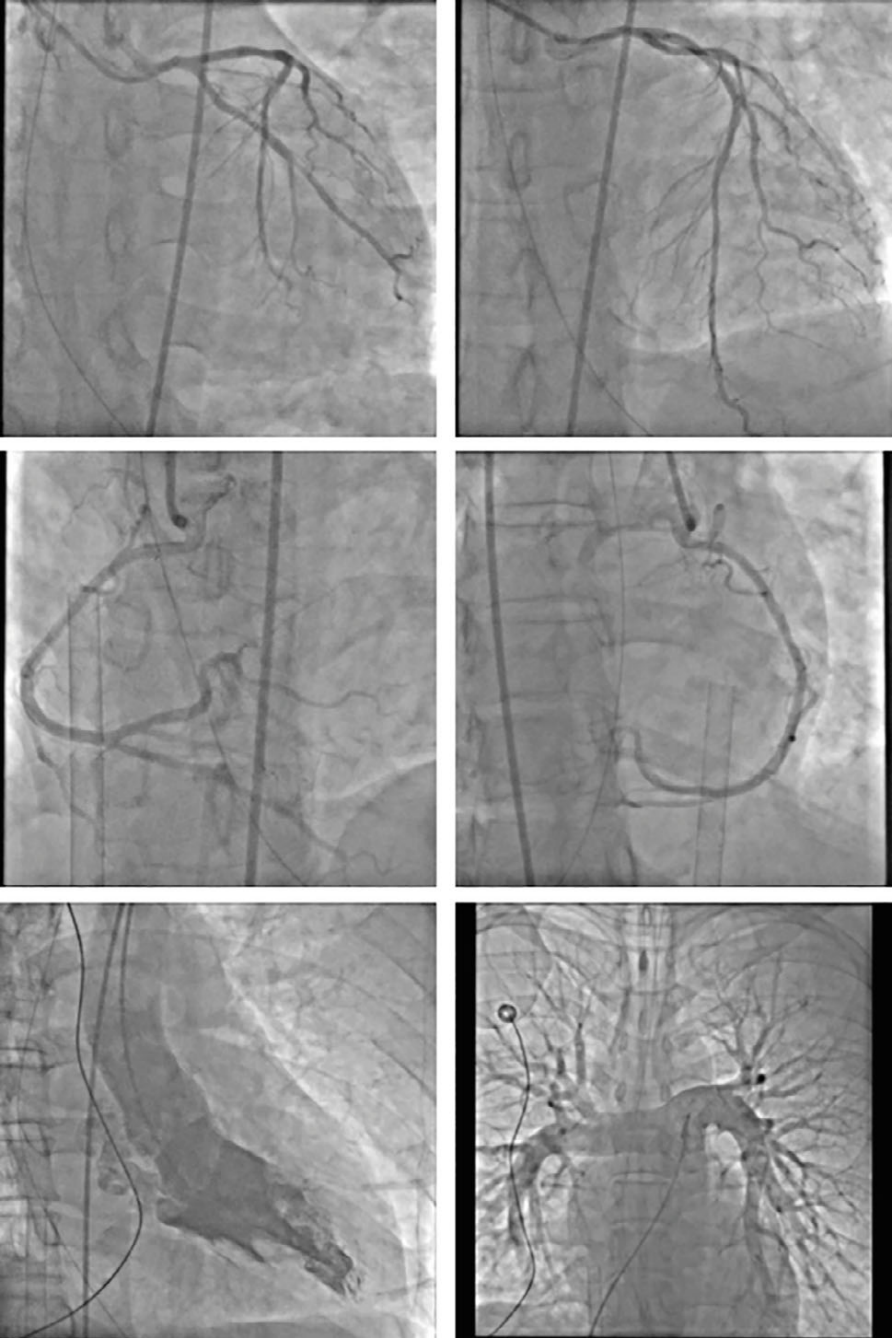
Coronary angiography demonstrated normal left main (LM) artery, 25% stenosis in the proximal left anterior descending artery (LAD), normal diagonal branch (D1), and 25% stenosis in the distal left circumflex artery (LCX). Left ventricular angiography and pulmonary artery angiography showed no significant abnormalities.

The patient, a 37‐year‐old male programmer in good health, was admitted to the Emergency Department of Nanjing First Hospital on July 19, 2024. At approximately 11: 10 on the same day, the patient suddenly experienced loss of consciousness and developed limb convulsions while at his workstation. His colleagues immediately began chest compressions. Upon the ambulance′s arrival, the patient′s electrocardiogram (ECG) remained a flat line. The emergency medical team immediately initiated continuous chest compressions using the LUCAS device and maintained bag–valve–mask ventilation without interruption from the time they arrived at the scene until the patient was transported to the hospital. The patient arrived at the emergency department of the hospital at 11:44 am. At that time, the patient was unconscious with dilated and fixed pupils and had no spontaneous breathing or palpable heartbeat, and the ECG displayed a flatline. We continued chest compressions using the LUCAS device, performed endotracheal intubation and mechanical ventilation, and administered 1 mg of epinephrine intravenously. End‐tidal CO_2_ remained between 25 and 30 mmHg, indicating a poor prognosis. At 11:59, the ECG showed ventricular tachycardia, and 0.1 g of lidocaine was administered intravenously. It remained a ventricular tachycardia rhythm. Consequently, venoarterial ECMO was initiated. ECMO was initiated at 12:28, with a centrifugal pump speed of 3500 rpm, an oxygen concentration of 100%, an airflow rate of 3 L/min, and a blood flow rate of ≈2.6 L/min. Owing to the pulseless ventricular tachycardia rhythm of the patient, multiple 200‐J biphasic defibril lation shocks were administered. Autonomous cardiac rhythm was restored at 13:13, and electrocardiography revealed premature ventricular contractions, QS pattern (V1 and V2), poor R wave progression (V3), significant ST‐T changes, and the troponin I was 0.113 ng/mL. Blood pressure was ≈120/80 mmHg while receiving norepinephrine at 2.0 *μ*g/kg/min. However, spontaneous breathing was absent; therefore, mechanical ventilation was continued. Given the unknown etiology of sudden death, emergency imaging studies including CT, coronary angiography, left ventriculography, and pulmonary angiography were conducted to identify potential underlying causes. Head CT revealed no evidence of cerebral hemorrhage but suggested possible brain swelling (Figure 1a). Coronary angiography demonstrated normal left main (LM) artery, 25% stenosis in the proximal left anterior descending (LAD) artery, normal diagonal branch (D1), and 25% stenosis in the distal left circumflex (LCX) artery. Left ventricular angiography and pulmonary artery angiography showed no significant abnormalities (Figure 2). So far, the exact cause of his sudden death has yet to be determined. Following the procedure, the patient was transferred to the intensive care unit (ICU) for continued monitoring and further treatment. Upon ICU admission, the patient was in a deep coma (GCS 2T, bilateral pupil diameter 2 mm with absent light reflex), tachycardic at 127 beats/min, and had an arterial blood pressure of 100/70 mmHg, which was maintained with epinephrine infusion at 0.01 *μ*g/kg/min and metaraminol at 10 mg/h. Blood gas analysis revealed a pH of 7.3, PCO_2_ of 34 mmHg, PO_2_ of 87 mmHg, HCO_3−_ of 17.7 mmol/L, base excess of −7.3, and anion gap of 30.5 mmol/L, indicating metabolic acidosis. His lactate was 14.18 mmol/L.The mechanical ventilation settings were as follows: synchronized intermittent mandatory ventilation (SIMV) mode, FiO_2_ of 60%, respiratory rate of 16 breaths per minute, pressure control at 20 cmH_2_O, positive end‐expiratory pressure (PEEP) at 8 cmH_2_O, and a monitored tidal volume of approximately 420 mL. Unfortunately, EtCO_2_ was not continuously monitored, which limited the ability to dynamically assess cerebral perfusion and metabolic status. The patient also received continuous venoarterial ECMO treatment, active brain protection via sedation and analgesia with midazolam at 5 mg/h and remifentanil at 0.08 *μ*g/min, combined with the use of an ice blanket and the ECMO heater to maintain a body temperature of approximately 35°C, as well as a negative fluid balance of 500–1000 mL/day for intracranial pressure (ICP) management. Due to the patient′s acute kidney injury (urea 6.68 mmol/L and creatinine 138.4 *μ*mol/L with oliguria) and metabolic acidosis, oXiris continuous renal replacement therapy (CRRT) was initiated at 15:00 to remove inflammatory mediators and stabilize the internal environment. Bedside echocardiography revealed a left ventricular ejection fraction of 21% and a cardiac output (CO) of 1.35 L/min, indicative of significantly reduced cardiac function. Additionally, the optic nerve sheath diameter (ONSD) was measured at 0.55 cm, with neuron‐specific enolase (NSE) levels at 137.3 ng/mL and S100 levels at 6.085 ng/mL.

Figure 1The patient′s head CT scan. (a) possible brain swelling upon admission (July 19th). (b) Diffuse cerebral edema along with localized ischemic–hypoxic encephalopathy (July 22nd). (c) Diffuse cerebral edema and intracerebral hemorrhage (July 24th). (d) No new hemorrhagic lesions or worsening of brain edema (July 26th). (e) Progressive resolution of the hemorrhage (July 30th).

**Figure:**
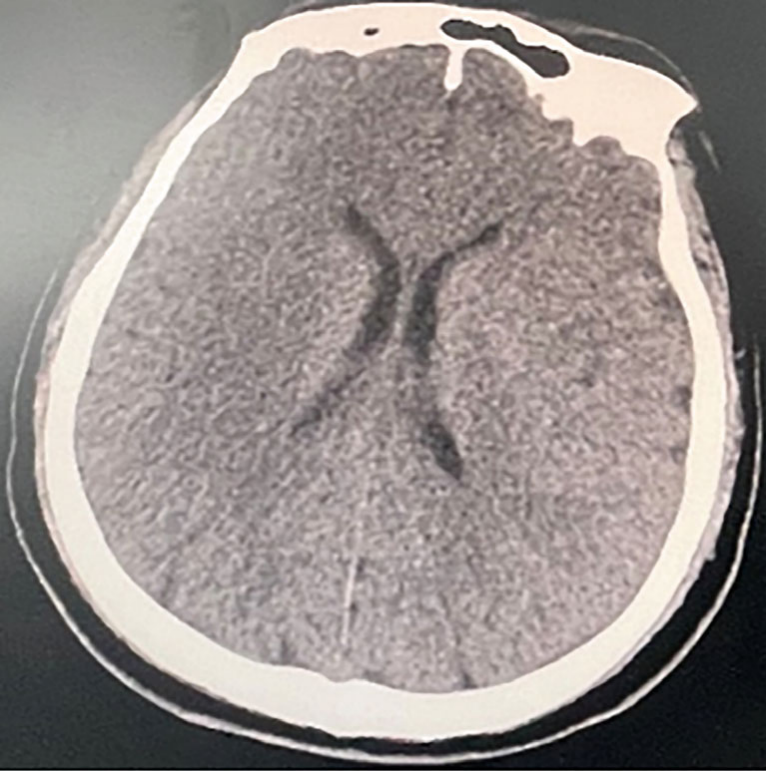
(a)

**Figure:**
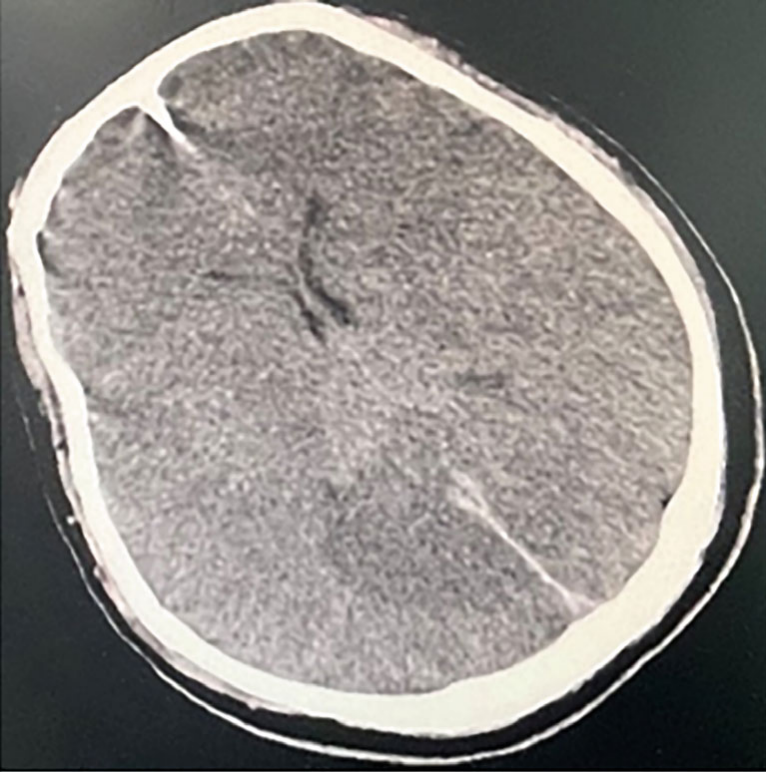
(b)

**Figure:**
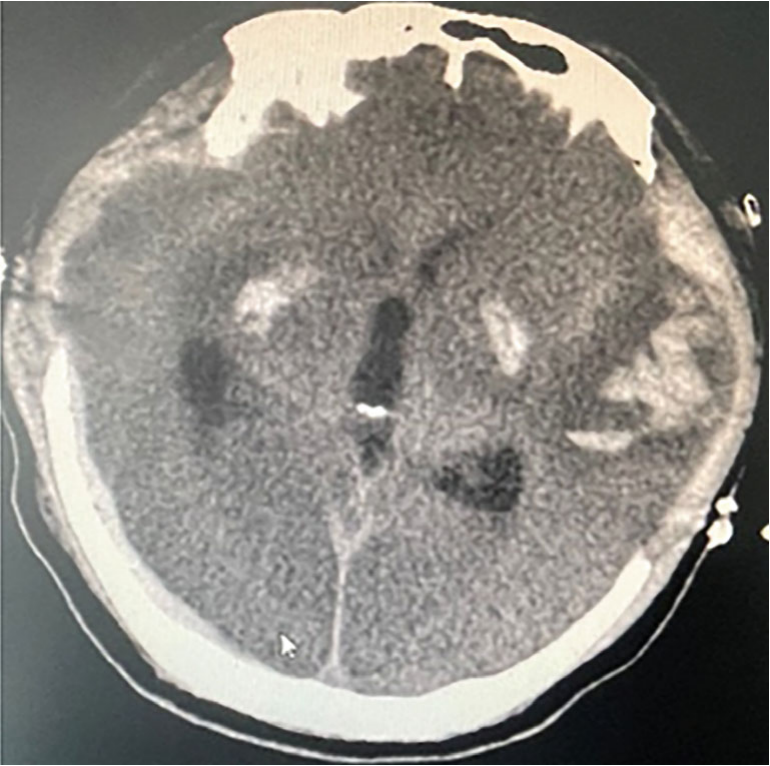
(c)

**Figure:**
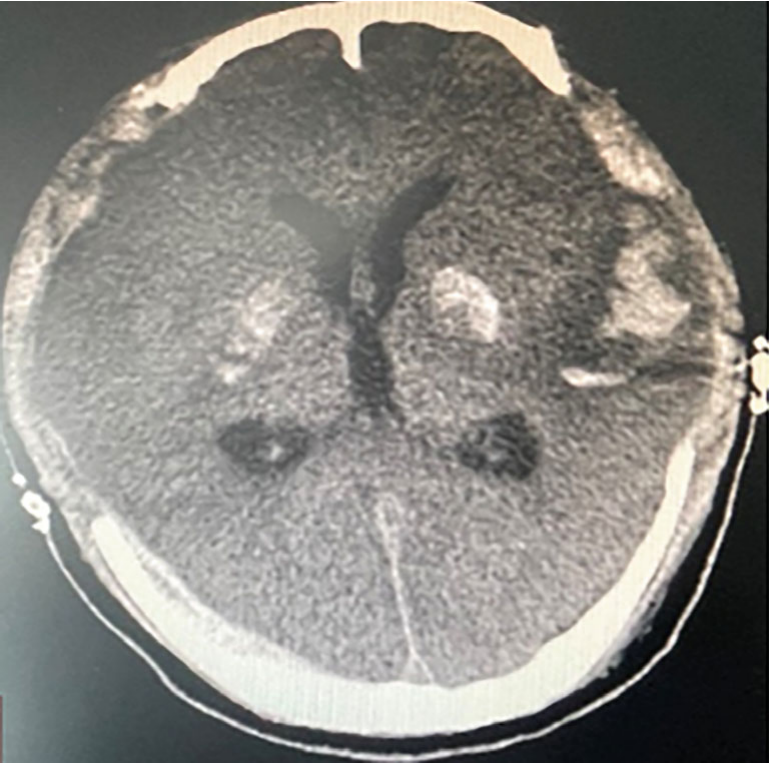
(d)

**Figure:**
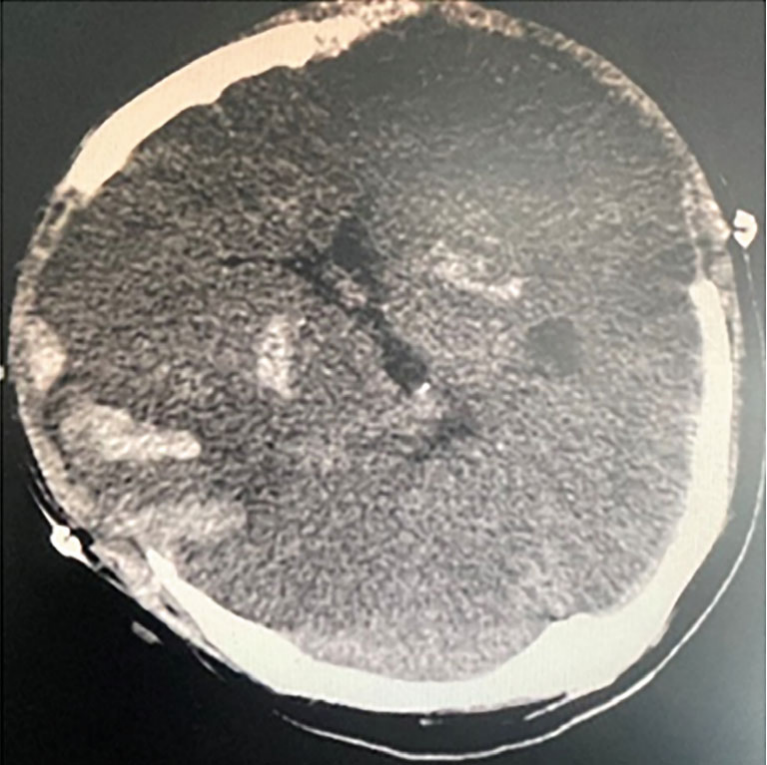
(e)

**Figure 3:**
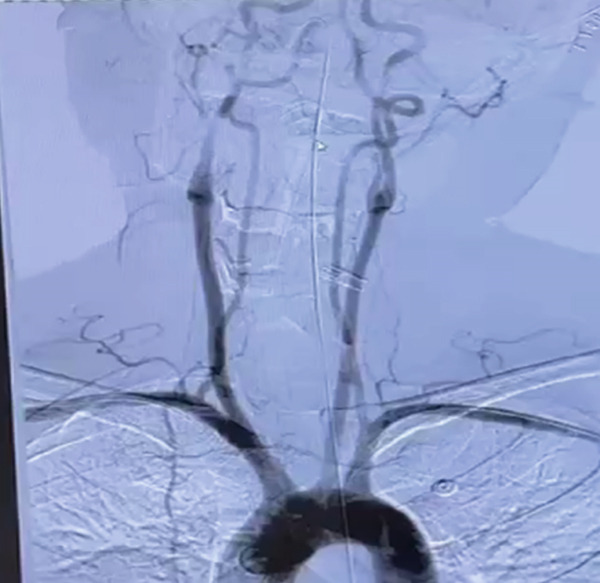
Cerebral DSA revealed cerebral venous sinus thrombosis (CVST).

The following morning during the ward round, it was noted that the patient′s bilateral pupils were dilated to 6 mm in diameter with absent light reflex, whereas they had been normal just half an hour earlier. We promptly conducted a head CT scan, which revealed diffuse cerebral edema along with localized ischemic–hypoxic encephalopathy (Figure 1b). Subsequently, we convened a consultation involving neurosurgeons from both within and outside the institution. The experts unanimously concluded that a bilateral decompressive craniectomy was necessary to reduce ICP; however, they also noted that the surgical risk was substantial. Despite this, the family expressed strong support for proceeding with the surgery. Following the signing of the informed consent form, it was decided to carry out the surgical intervention. The “bilateral decompressive craniectomy” was successfully performed under ECMO support. During the procedure, heparin anticoagulation was temporarily suspended, and the ECMO flow rate was maintained at 4 L/min. The total duration of the operation was 5 h. However, postoperatively, the patient′s pupils remained dilated without any signs of constriction. Follow‐up CT imaging revealed diffuse cerebral edema and intracerebral hemorrhage (Figure 1c). We once again convened a multidisciplinary consultation involving experts from the neurology and neurosurgery departments of the brain hospital, during which a cerebral digital subtraction angiography (DSA) examination was recommended to evaluate the status of cerebral arteries and veins. Following informed consent from the family, the cerebral DSA examination was promptly performed. Cerebral DSA revealed cerebral venous sinus thrombosis (CVST) (Figure 3). Treatment was initiated with continuous infusion of low molecular weight heparin at a dose of 5000 IU once daily.

Following treatment, the patient′s hemodynamics gradually stabilized. By July 24, norepinephrine was discontinued to 0.1 *μ*g/kg·min, and the left ventricular ejection fraction improved to 38%. So, the ECMO tube was removed on the same day. By July 28, the patient′s pupils had decreased in size to 4 mm on the right and 3 mm on the left. The ONSD was measured at 4 mm, with a peak systolic velocity of 100.40 cm/s, an end‐diastolic velocity of 42.52 cm/s, a resistance index (RI) of 0.57, and a pulsatility index (PI) of 0.94. The follow‐up CT scan revealed no new hemorrhagic lesions or worsening of brain edema (Figure 1d). These findings suggest no significant increase in ICP or abnormality in cerebral blood flow dynamics. Furthermore, the concentration of NSE progressively decreased from 65.55 ng/mL at 24 h post‐CPR to 13.5 ng/mL at 96 h, while the S100B concentration declined from 100 to 23 ng/mL during the same period. The patient exhibited spontaneous breathing; however, due to an inability to fully wean from the ventilator support, a percutaneous tracheostomy was performed on July 29. By August 1, the patient had achieved hemodynamic stability, with vasoactive drugs discontinued. Spontaneous breathing was slightly tachypneic but allowed for intermittent weaning from mechanical ventilation. Echocardiography demonstrated an improved left ventricular ejection fraction of 56%, and the patient′s level of consciousness had enhanced, reflected by a GCS of 5T (M2VTE3). CT demonstrates progressive resolution of the hemorrhage (Figure 1e). However, renal function remained impaired, necessitating continued intermittent dialysis. On that day, the patient was transferred to a rehabilitation hospital for hyperbaric oxygen therapy.

## 3. Discussion

Refractory cardiac arrest (RCA) is defined as the failure to restore spontaneous circulation (ROSC) despite 30 min of appropriate CPR [6]. Despite advancements in prevention, intervention, and advanced cardiac life support (ACLS), RCA remains a leading cause of mortality globally, with most patients failing to survive [7]. Over the past decade, ECMO‐based resuscitation has demonstrated promising outcomes in both in‐hospital cardiac arrest (IHCA) and out‐of‐hospital cardiac arrest (OHCA) scenarios, as well as in adult and pediatric populations [8]. Consequently, ECPR is increasingly utilized as an attempt to improve survival and prognosis in these challenging cases [5]. The optimal timing for initiating ECPR remains unclear. However, prolonged low‐flow periods are generally associated with poorer prognoses. Studies indicate that after 30 min of conventional CPR, the survival rate with favorable neurological outcomes is typically below 1% [9]. Consequently, resuscitative efforts are often discontinued in patients experiencing excessively long low‐flow durations. Notably, recent case reports have highlighted instances where ECMO support was successfully initiated following prolonged CPR lasting 95 and 82 min, respectively, resulting in favorable neurological recoveries [10, 11]. The common features of these successful cases include continuous chest compressions performed by bystanders, the use of the LUCAS device to maintain compressions upon arrival at the hospital, and the presence of potential life signs in patients (e.g., ventricular fibrillation rhythm or gasping). Studies have demonstrated that as vital signs improve, the likelihood of a favorable prognosis also increases [12]. This finding aligns with prior reports. A recent study further confirmed that gasping during transport is significantly associated with favorable neurological outcomes in patients receiving ECPR, likely because gasping directly and partially reflects brainstem activity during CPR [13]. High‐quality chest compressions are a critical component of the CPR survival chain during CA, as they directly influence hemodynamic stability and neurological outcomes [14]. In this case, the patient′s CA was witnessed by colleagues who had all undergone professional medical training. Consequently, they promptly initiated chest compressions and delivered high‐quality CPR. This case clearly highlights the essential role of widespread CPR training in enhancing the survival rates of individuals experiencing CA. Upon arrival, the emergency physician utilized the LUCAS device to perform continuous chest compressions. This mechanical compression device is capable of delivering consistent, high‐quality compressions without interruption, even during patient transport. High‐quality, uninterrupted compressions provided by such devices have been shown to be associated with improved clinical outcomes and prognosis [15]. Recent reports of successful rescues following prolonged high‐quality CPR and subsequent ECPR have consistently shown that patients presented with ventricular fibrillation and low blood flow upon arrival at the emergency department. In contrast, in our case, the patient exhibited asystole upon arrival and did not develop ventricular tachycardia until 15 min later, resulting in a no‐flow period lasting 59 min. Therefore, even when CA persists beyond 30 min, ECPR should still be actively considered for young patients under the ongoing support of continuous CPR.

In addition, addressing the underlying causes of reversible CA is critically important [16]. The etiologies of cardiac and respiratory arrest are commonly summarized using the 6H6T5C mnemonic, which is outlined in Table 1. A primary function of ECPR is to provide a time window for effectively treating these reversible causes [17]. In cases of CA attributed to suspected acute myocardial infarction (AMI), ECPR sustains blood pressure and oxygenation, providing a critical time window for emergency percutaneous coronary intervention (PCI). PCI involves stent implantation to relieve coronary artery occlusion, restoring myocardial perfusion and improving the chances of patient survival [18]. ECPR was implemented in two patients who experienced CA secondary to embolic events (one due to pulmonary embolism and the other due to amniotic fluid embolism). With aggressive anticoagulation therapy, both patients successfully stabilized. This outcome can be attributed to ECPR providing a critical time window for addressing the underlying conditions; otherwise, early ischemia and hypoxia could have rapidly led to fatal outcomes [19, 20]. In a greater number of cases, ECPR was utilized in patients experiencing CA secondary to fulminant myocarditis. ECPR maintained systemic circulation and tissue perfusion, providing a critical time window for resolution of myocardial edema and recovery of cardiac function. Ultimately, patients achieved complete recovery without any residual sequelae [21, 22]. Our medical records indicate that the patient experienced initial pupil dilation following ECPR, with CT findings revealing diffuse cerebral edema. To address the cerebral swelling, a “bilateral decompressive craniectomy” was performed under ECMO support without anticoagulation and at a high flow rate of 4 L/min, lasting for 5 h. This case highlights an uncommon heparin‐free treatment approach during ECMO therapy in the absence of contraindications to anticoagulation. There have been limited case reports on decompressive craniectomy performed under ECMO support, with existing literature predominantly focusing on conditions such as brain abscess, cerebral hemorrhage, and extensive cerebral infarction [23–25]. To date, no reports have specifically addressed the implementation of this surgery for patients with diffuse cerebral edema in the context of ECPR. Unfortunately, 24 h postoperatively, the patient′s pupils remained dilated. Follow‐up head CT demonstrated diffuse cerebral edema and intracranial hemorrhage, while cerebral angiography confirmed thrombosis of the intracranial venous sinuses.

**Table 1:** The causes of cardiac and respiratory arrest‐6H6T5C.

**6H**	**6T**	**5C**
1. Hypothermia	1. Coronary thrombosis	1. Cerebral causes
2. Hypoxia	2. Pulmonary thrombosis	2. Cardiomyopathy
3. Hypovolemia	3. Cardiac tamponade	3. Conduction abnormalities
4. Hypoglycemia	4. Tension pneumothorax	4. Congenital abnormalities
5. Hydrogenion (acidosis)	5. Trauma	5. Commotio cordis
6. Hyper‐/hypokalemia	6. Toxins	

**Figure 4:**
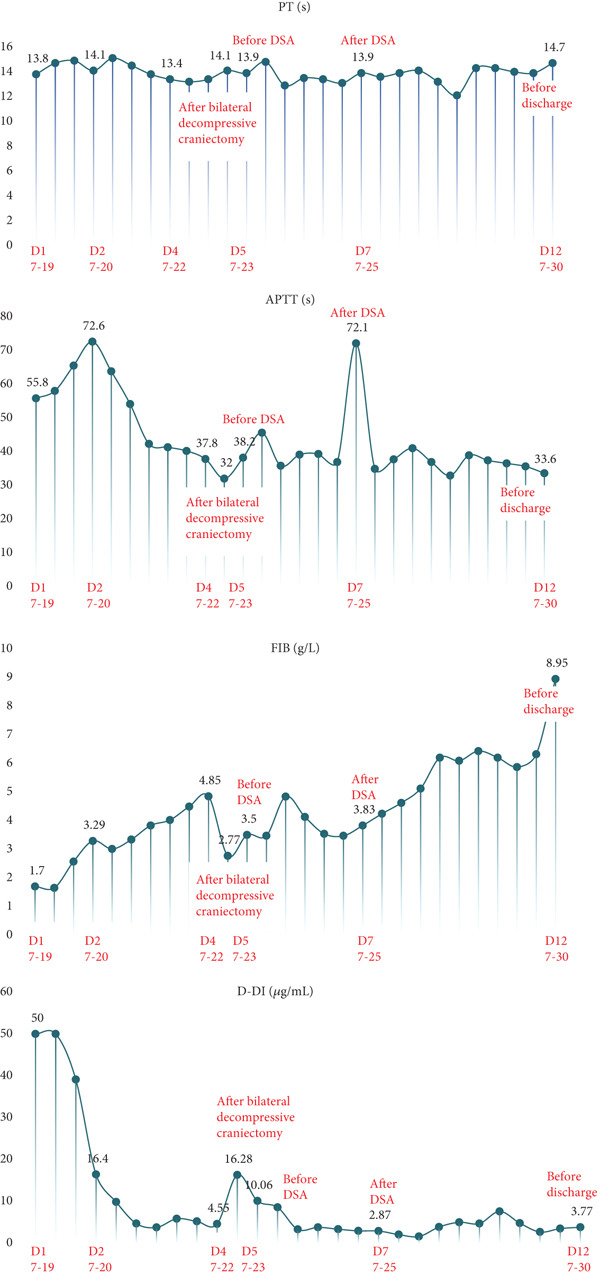
The changes in the patient′s coagulation indicators (PT, APTT, fibrinogen, and D‐dimer).

CVST is a cerebrovascular disorder caused by thrombus formation in the intracranial veins or venous sinuses due to various etiologies. This condition impairs venous outflow and cerebrospinal fluid circulation, resulting in intracranial hypertension and focal brain injury. CVST constitutes approximately 0.5%–1.0% of all cerebrovascular diseases. The causes and predisposing factors of CVST include genetic factors, infections, autoimmune diseases, medications, and iatrogenic factors. Then, 90% of patients experience headaches, while 40% exhibit focal or generalized epileptic seizures, along with increased ICP and motor or sensory dysfunction. DSA is considered the gold standard for diagnosing CVST, as it clearly demonstrates complete venous sinus occlusion by thrombus, resulting in the characteristic “empty sinus sign.” Anticoagulation remains the primary treatment for CVST. In cases where patients are comatose and their condition is progressively worsening, thrombolytic therapy or mechanical thrombectomy may be considered. For patients at risk of developing brain herniation, decompressive craniectomy is recommended as a life‐saving intervention to prevent mortality [26]. Our patient was ultimately diagnosed with CVST; however, it remains unclear whether this condition precipitated the CA or occurred secondary to it. A retrospective analysis of the patient′s coagulation profile offers valuable insights. As shown in Figure 4, D‐dimer levels were markedly elevated upon admission, accompanied by decreased fibrinogen, findings consistent with disseminated intravascular coagulation (DIC) following CPR. With aggressive correction of coagulopathy, D‐dimer levels declined and fibrinogen recovered. Notably, on July 23,following bilateral decompressive craniectomy, D‐dimer levels sharply increased again, while fibrinogen remained within the normal range, suggesting active thrombus formation. This hypercoagulable state coincided with the perioperative interruption of anticoagulation, a hypothesis further supported by thrombus formation observed in the ECMO circuit postoperatively. This period likely represents the onset window for CVST, which was subsequently confirmed by DSA. Following the initiation of anticoagulant therapy, D‐dimer levels gradually declined, ECMO support was successfully weaned. The patient exhibited marked improvement in cardiopulmonary function and partial recovery of neurological function, ultimately leading to successful discharge. Taken together, the temporal pattern of coagulation markers suggests that CVST was likely a consequence of the prothrombotic state induced by anticoagulant withdrawal, rather than the primary cause of CA.

What is the relationship between intracranial venous sinus thrombosis and CA? A review of the literature reveals potential bidirectional causality. In 2023, Al‐Attas et al. reported a case of a 28‐year‐old woman with extensive CVST leading to elevated ICP, which in turn induced sinoatrial node dysfunction (SSS), manifesting as a prolonged sinus pause of 9.28 s. The patient survived following emergent placement of a temporary pacemaker, and the authors explicitly attributed the severe bradycardia to “increased ICP–mediated excessive vagal excitation” [27]. Another case described a child with closed head injury complicated by superior sagittal sinus thrombosis who suffered sudden CA 6 h postoperatively and could not be resuscitated. Retrospective imaging analysis confirmed that diffuse cerebral edema and brainstem compression secondary to venous sinus thrombosis were consistent with neurogenic CA [28]. Conversely, a Chinese case report from 2009 documented a patient who experienced CA due to acute pulmonary embolism and died after multiple unsuccessful CPR attempts. Postmortem examination revealed thrombosis in the sagittal sinus and bilateral sigmoid sinuses, leading the authors to propose that recurrent CA and associated hemodynamic instability contributed to the development of intracranial venous sinus thrombosis [29]. This may be attributable to the “low‐flow, reperfusion‐induced hypercoagulable state” following CPR, which can promote the development of CVST. Collectively, these case reports suggest a potential bidirectional relationship between intracranial venous sinus thrombosis and CA—wherein each condition may precipitate or exacerbate the other. However, the precise underlying mechanisms remain poorly understood and warrant further investigation through large‐scale, systematic studies.

## 4. Conclusion

The use of ECPR in conjunction with traditional high‐quality CPR can significantly enhance the survival rate and neurological recovery outcomes for patients experiencing CA. For younger patients with CA, ECPR should be considered more proactively rather than being strictly constrained by time limitations alone. Intracranial venous sinus thrombosis and CA may have a bidirectional causal relationship. Clinicians should remain vigilant for the occurrence of this potentially life‐threatening interaction. This successful case demonstrates that timely high‐quality CPR performed by bystanders, prompt initiation of ECPR, and subsequent comprehensive management of the underlying cause are critical factors in improving survival rates and achieving favorable neurological outcomes for CA patients.

## Conflicts of Interest

The authors declare no conflicts of interest.

## Funding

No funding was received for this manuscript.

## Data Availability

This is an open access article distributed under the Creative Commons Attribution License, which permits unrestricted use, distribution, and reproduction in any medium, provided the original work is properly cited.
